# Supplementary material for: Patterns of Intron Gain and Loss in Fungi
Source: PLoS Biol. 2004 Nov 30;2(12):e422. doi: 10.1371/journal.pbio.0020422 (PMC532390; doi:10.1371/journal.pbio.0020422)
Supplement: Table S1 — Also available at http://genes.mit.edu/NielsenEtAl/. (4.3 MB ZIP). [file pbio.0020422.st001.zip › NielsenEtAl/html/961.html]

AN0576.1.NCU06626.1.MG06100.1.FG05306.1


```
 CLUSTAL W (1.82) Multiple Sequence Alignments - Introns Inserted


Sequence 1: NCU06626.1	1563 aa
Sequence 2: MG06100.1	1568 aa
Sequence 3: FG05306.1	1525 aa
Sequence 4: AN0576.1	1599 aa
Alignment Length: 1679 aa
Number Identitical Residues: 548 aa
Alignment Score (without introns) 31914


MG06100.1 	MGQGLSLTSPPAGSAGIDIPELGDLVYEKSVGTARFLKCIRARHHDGVVLVKVLTKPYTP
NCU06626.1	MGQGFSLTAPPAGAAGIDVPELADLTYEKVIGQARFMKSIRARHNDGVVLVKVLVKPYA-
FG05306.1 	MGQGFSLATPSVGSAGIDISELSDVQYEKSIGNARFMKTIRGRHENGLVLVKLLVKPYPE
AN0576.1  	MGQGYSLTTLSAGSAGIDIPELSDLTYEKSMGGGRFMKSIRARQKNGLVFVKVIMKPYPS
          	**** **:: ..*:****:.**.*: *** :* .**:* **.*:.:*:*:**:: ***. 

MG06100.1 	MPLDDYRQEIIR1ESKALADVPNALAYQRVIETDTNGYLVRQYLYNSLYDRFS~TRPFLE
NCU06626.1	MSLGRFKKKIIR1ERNALASVPNALPYQRAIETETNGYLVRQFFYNSLYDRLS~TRPFLE
FG05306.1 	VKLEKYKKQIIE1QHKAIADVPNALGFQRIIETESNGYLVRQFLYSSLYDRLS~TRPFLE
AN0576.1  	MKLEPYVRAIIR1ERKLLSDIPNALAYQRILETSTGGYLVRQYIHSSLYDRMS2TRPFPE
          	: *  : : **. : : ::.:**** :** :**.:.******:::.*****:* **** *

MG06100.1 	DIEKKWLAFQLLCALRDCHSRDIYHGDIKTENTLVTSWNWLYLSDFSSSFKPVILPDDNP
NCU06626.1	DMEKRWLAFQLLCALRDCHAKEIYHGDIKTENTLVTSWNWLYISDFSSSFKPVMLPEDNP
FG05306.1 	DIEKKWLAFQLLCALRDCHARDVYHGDIKAQNVLVTSWNWLYLSDFSSAYKPVMLPDDNP
AN0576.1  	DIEKKWIAFQLLCALRDCHAVDVFHGDIKTENVLVTAWNWVYLSDFSSSFKPTFLPEDNP
          	*:**:*:************: :::*****::*.***:***:*:*****::**.:**:***

MG06100.1 	ADFSYFFDTSGRRTCYIAPERFQPAGDALNSPIKLNWAMDIFSAGCVIAELFTEAPIFSL
NCU06626.1	ADFSYFFDTSGRRTCYLAPERFLPPGQDPDPNAKITWAMDVFSAGCVIAELFLETPIFNL
FG05306.1 	ANFSYFFDTSGRRTCYIAPERFYASGEAPPQKAKMTWAMDIFSAGCVIAQMFLESEMFSL
AN0576.1  	ADFSFYFDTSGRRTCYLAPERFLVANEEPGS-RNVNWAMDIFSAGCVIAELFLESPIFTL
          	*:**::**********:*****  ..:      ::.****:********::* *: :*.*

MG06100.1 	SQLYNYRSGKYDPAISHLSHIPDKDLREMIIHMIQLDPQKRYSAEDYLRFWRKTVFPDYF
NCU06626.1	SQLYKYRRGEYDPSISHLSRIPDKDLREMIAHMIQVDPQKRYSAEQYLDFWKDKVFPGYF
FG05306.1 	AQLYKYRRGEYDPVITHLSVIADKDIADMIAHMIQLDPEKRYSAEQYLDFWKGKVFPHYF
AN0576.1  	SQIYKYRKGEYSPEHSQLAKIEDPEIRALILHMIQLDPESRYSAEEYLNFWKIKAFPEYF
          	:*:*:** *:*.*  ::*: * * ::  :* ****:**:.*****:** **: ..** **

MG06100.1 	YDFLHQYMEVITDPSSGQYPISGATRNLGEADERIDRVFYDFDKISYSLGYQHNASSGNG
NCU06626.1	YNFLHQYMQSITDLSTG-------NSRIGEADQRIERVWNDFDKISYFLGYANGNDPYEH
FG05306.1 	YNFLHQYMGLITDPSSGNNPMSGASKNLGEADERIDRVFYDFDKISYFLGYQLEKRTSQE
AN0576.1  	YSFLHQYMSLMTDPSSGRTQVEAGSANRGEADDRIERVYLDFDKISSLLGAHLEPGKDGS
          	*.******  :** *:*    .... . ****:**:**: ******  **          

MG06100.1 	ALVPKPPRLGLGHFPIRLNIPNHEHYVSSQGQPPSDDGSLIFLALICSSIRNTARAASKI
NCU06626.1	HLPS--PRLGLGLFPVRLNVPNAEHHVSANKQPATDDGTLIFLTLVASSLRNTAHATAKI
FG05306.1 	FKPS--SRLSLSHFPVRLNIPNHNHTVSAALEPPEDDGTLIFLTLIVSQLRNTARASSRI
AN0576.1  	LSTT--SKLTSGTFPVELDLPRYKYSDRTS----TDDGVLIFLTLVVSSLRSTSKSSARI
          	   .  .:*  . **:.*::*. ::   :      *** ****:*: *.:*.*::::::*

MG06100.1 	RACDILLAFSERITDEAKLDRVVPYLMTLLN-DKADMVVITAIRTITQVLALVKVVTPVN
NCU06626.1	KACDIMLAFAERLTDEAKLDRVLPYLMTLLT-DKCDRVAISALRTITQLLDLVKIITPVN
FG05306.1 	RACDVLLAFSERLTDEAKLDRVLPYLMTLLVPYETDLVLIAAIRTITQLLELVKMVTPIN
AN0576.1  	KACDILLAFAERLSDEAKLDRILPYIMILLN-DRTDSVKIAAIRSLARLLEMVQVVSPVN
          	:***::***:**::*******::**:* **   . * * *:*:*:::::* :*::::*:*

MG06100.1 	SPIFLEYILPRMQVALTSAQANPSPLVRMTYASCLGTLATTAGRYLDIAGALRAEGAITV
NCU06626.1	SHIFLEYILPRMQVALLGSQGTPSPVVRATYAACLGKLATTAYRFLAMAATLRADGSVTI
FG05306.1 	SHVLVEYVLPRMEIALSSRVRTPSPLVRAAYASCLGSLATTAQRFLNMASSLRPDGSIPI
AN0576.1  	AYLFSEYIFPRLQPFVPNANSQPSPAVRAAYASCIASLAHSSLRFLDMIQALRSDTRLPA
          	: :: **::**::  : .    *** ** :**:*:..** :: *:* :  :**.:  :. 

MG06100.1 	ADPE-VEPG--STTAFDGMFEHTRHELVIQFELHVKLLIEDPDPFVRRAFLSSVPELCMF
NCU06626.1	SDPE-LEPGNETNTALDGLFDSSRQDLVDLFETHTKMLIEDLNPFVRRAFLSSVPDLCIF
FG05306.1 	SDPE-VEPGADAKANFESVFDNAGRQLFEVLESHTKQLVEDSDNSVRRAFLASVPELCYF
AN0576.1  	LIPAGSEPRWTEDSTYHNLYDVARIDLLDYFETHTKALLTDTDASVRRAFLGSVSSLCVF
          	  * . **     :  ..::: :  :*.  :* *.* *: * :  ******.**..** *

MG06100.1 	FGTADSNDIILAHLNTYLNDRDWTLKCALFDTIVGIAAFIGSTSLDEFILPLMVQALMDT
NCU06626.1	FGVAKANDVIIAHLNTYLNDRDWMLRYAFFDTVVGISAFMGSSTLESFILPLIIQAVDDP
FG05306.1 	FDDH-SNDVLLTHLNTYLNDRDWTLKCAFFDTIVGIAIFVGSTSLEEFMLPLMIQALADT
AN0576.1  	FGTIKTNEVILSHLNTYLNDRDWILKCAFFEAVVGVAVYVGTTSLEQYILPLMVQSMTDP
          	*.  .:*:::::*********** *: *:*:::**:: ::*:::*:.::***::*:: *.

MG06100.1 	EENVVQGALHSLAELAELGLLSKSRVCELVEVVARFTMHPNIWIRESATEFIAMATKYLT
NCU06626.1	EEFVVQGALQSLAELTRLGLLTKEAFVDLVALVRRFTMHPNIWIREAAAHFIAAGNEFLS
FG05306.1 	EENVVQAALHSLAQLARLGLLSKPKIWELVDLISRFTMHPNLWIRESAAEFLSESARFLE
AN0576.1  	EEFVVERVLRSLAAMADLGLFQRPTTWDLLHATVRFLVHPNAWIREAAVSLVVNSTKFLS
          	** **: .*:*** :: ***: :    :*:    ** :*** ****:*. ::  . .:* 

MG06100.1 	AADIRCMVYPIVTPYLKPGITPNFSELALLGTLRRPLTRPVFDQALLWALKSDRGLFWKP
NCU06626.1	SAYLKVSVYPQLKPFLKPDIIPGWSELQLLDSLQKPLSRNVFELAMQWAAKSEKSGFWKP
FG05306.1 	PADVRCNLMPLVKPFLKIEVLSDFQELTILDALKRPLTRNVFDQAITWALKTERGVYWKP
AN0576.1  	TADKYSILTPLVRPFLKVSIVD-FSEGDILDALKTPLPKALYDQAFVWASQAEKGFFWKS
          	.*     : * : *:**  :   :.*  :*.:*: **.: ::: *: ** ::::. :**.

MG06100.1 	LQKARALMLGNSPAATGNGRGSSEHNPGTLGKVARNEEDEQWLSRLRNLGLSQEDEHKLL
NCU06626.1	SKR-----LWDARAVT---------------KLSKTDEDEQWLGRLRTAGLSQADEMKLI
FG05306.1 	LQQLKPLLFGYKGSAS--SRSSRELAQGSMGRVARNDEDEQWVAKLRNLGLKHEDELKLD
AN0576.1  	VGRDGASLGSVDAAFQ-----KRASKAFSLSAQPKNDEDEQWIAKLRALGLTTEDESKLL
          	  :  .       :       .      : .  .:.:*****:.:**  **.  ** ** 

MG06100.1 	SLREFIWRLSQMKTRDSASARDEDKTSKQLNNIILLRTLGVTPQMVFFD-----------
NCU06626.1	MLREFIWRLVQMKGRDSTELQMEKRS--ELEDIIPLRNLGITPQTVMFE-----------
FG05306.1 	ALREFIWRLSKMKARDASTADGSTSN-----GVVSLKTLGVNPQTVFFN-----------
AN0576.1  	ALREHIWRVSMRQTKDHESAGASLNN------VVALIDYGVTPQTVFFDKSQNVKARRNH
          	 ***.***:   : :*      .  .      :: *   *:.** *:*:.:.. .:  . 

MG06100.1 	EEPKGLSQ-GGGEL-DGAPRTITDALRDASMSIDDPVARRRRAALNSHRTRLNSRNSLSP
NCU06626.1	EEPIRYPAPQLDDD-DTAPKTIQEALLDASLTIGDPLSQRRRAAVDKQKSKSGSRSNLPS
FG05306.1 	DAPLRDPKAVVDLERPEEPYTIADALLDASMTIDDSTGGKRKANTNRRVQSVGPRS----
AN0576.1  	ENPVGRPQLTRAED--SKAHTIADALLDASTTIDNNANSRRRHARTKSQAQQEIEAAIST
          	: *   .           . ** :** *** :*.:    :*:            .   ..

MG06100.1 	MSIEGRMSLDDEMAG--------APSSTSPGLLSPPGD-------------------GEK
NCU06626.1	ISVDGRLATDSNGEGSRDTLRRSASHQSRASLLSPHDD-------------------VGS
FG05306.1 	---SGRFLSPTTAAR-------THSRTASASHAGYTGD-------------------DGS
AN0576.1  	PRPNNIEVLRAEDSPASSPIASSPSAAPGSRPMSPPSSDAERKLPEGRRSPGQESSSTET
          	   ..          : ..   : .  . .   .  ...:. . ...  :....:::  .

MG06100.1 	HRSNGQGPKRTLRHQSSALSLLNRKDNAKSGAETGMTEANAFGQVGGPSSQASKNKPLAA
NCU06626.1	AHYSPYETRRALKHQSSAMSLMNRKDSAKSVPETGTTETNAFGEVEAPLSQHYQGTHIRS
FG05306.1 	TQESSLS-RSGIRHQASALSLLDRKDKNKSIAQTGTTDTNAFGEVEGPFAQASAEQLTSS
AN0576.1  	DNLSLRRLKAGVQKKSSAISLLNRRDTAKAYAETGTSSANVFGKIGVPAQRDAISPAPRE
          	 . .    :  :::::**:**::*:*. *: .:** :.:*.**::  *  :         

MG06100.1 	DAAEDPLAKN--RPRANHSYEGNDPNILRMLDSMYVDNYPHDVAEFGPLVAPIARRRVSR
NCU06626.1	PDTESVPPTPGIKLRVNHDYHGNDPHILKMLDTMYVENYPHDLAEFGPEVTPIGRRKTGK
FG05306.1 	NGKEGDTVRP---RVSKHSYEGNDPNIQRMLDNMYVDNFPRDVIEFGPIITPIKRSKASR
AN0576.1  	RKPVDLQTHQ---YRANHSYAGNDPMILRLLDSVFAENYPTDFFDLGPYVKELDTRRPIM
          	    .           :*.* **** * ::**.::.:*:* *. ::** :  :   :   

MG06100.1 	PATADGSEQ---GWRPAGKLVATFSEHTGAINRVVPSPDHVFFITGGDDGTVRVWDTARL
NCU06626.1	IISSQPGKQRENGWKPKGVTIASFVEHVGAINRIVVAPDHQFFLTGGDDGTVKVWDSARF
FG05306.1 	VSIQPREEP----WKPEGQLVATFAEHKSAVNRVLPSPDHVFFITGGDDGTVRVWDTARL
AN0576.1  	KADGDVSKV----WKPTGTLVTLFGEHSGPVNRVVVAPDHSFFITGSDDGTVKVWDTTRL
          	       :     *:* *  :: * ** ..:**:: :*** **:**.*****:***::*:

MG06100.1 	ERNIAHRSRAVYSHAPGVRVLSLCFIQDTHCFVSCGSDGSVHIVKVECSMSGDVTRYNKQ
NCU06626.1	EKINIHKPRLTHRHAPGAKVRALCFIENTHSFVSCATDGSVHIVKVETYLQGESYRYTR-
FG05306.1 	ERNITHRSRQVHKHGDNTQVVALCFVENSHCFVSCASDGSVHVVKVDTASASGVVRYGK-
AN0576.1  	EKNLTPRSRQTYRHSSDAKVRALTFVENTHTFVSGATDGSIHAVKVGYHNSNGTVRYGK-
          	*:    :.* .: *. ..:* :* *::::* *** .:***:* ***     .   ** : 

MG06100.1 	PRMIRRYQLNRP----GEFAVWCEHYKQQGNGSPGSTEGGSTGFNSTTSTLILATNMSRV
NCU06626.1	LRTVREHRFVD-----GEFGVWCEHYKRDNE-----------------SILVVATNKSVV
FG05306.1 	LRVLREYQLPD-----GEFAVWCEHFRQESN-----------------SVLIVATNRSRI
AN0576.1  	LQLVREYQLSTTDDASPEYAVWMEHFRTDAQ-----------------STLLIATSMCRI
          	 : :*.:::  ...:: *:.** **:: : :                 * *::**. . :

MG06100.1 	EAIDLRNTTLIWALENPVHHGTPTCFCLDRKRNWLCLGTSHGVLDLWDLRFKVRLKAWGV
NCU06626.1	WGIELRTMTLLFKLENPVHHGTPTCFCIDFKKNWLCLGTSHGVLDLWDLRFKVKLKSWGV
FG05306.1 	LGLDLRTMDLLYTLENPVHHGTPTCFCVDRKRNWLCVGTSHGVVDLWDLRFKMRLKGWGL
AN0576.1  	IALDMKSMRPVYTLQNPTHHGTPTSFCCDRKHNWLLVGTTHGILDLWDLRFQVRLKAWGL
          	 .::::.   :: *:**.******.** * *:*** :**:**::*******:::**.**:

MG06100.1 	PGKGAVYRLAVHPTKGRGRWVCVAGGTGQG-EVTVWDLEKTTCREVYRVGG---------
NCU06626.1	PGKGSIYRLAIHPSRGRGRWVCASGGTGQG-EVTVWDIEKATCREVYRVGG---------
FG05306.1 	PGKGSIYRICIHPTKGRGKWVCISGGTGQG-EVTVWDLEKTVCREIYRAGG---------
AN0576.1  	SGSGPIHRLQLHPTKGHGRWVCVSASGNHGNEIIVWDIEKTKCREVYRADSPALGHNHPN
          	.*.*.::*: :**::*:*:*** :.. .:*.*: ***:**: ***:**....: . . ..

MG06100.1 	-------------NKEAPRDYSAWEVDEDKPEGMLGRFATDIETGEAPHVTGGSDRGVRA
NCU06626.1	-------------NKEGPKGYDAWQVDEDKPSDGQGQMLSRFATNIEPNAMGNADRGVRA
FG05306.1 	-------------SKDGLKGYEAWDVDEDKREGMLG----RYATDIEPSETANADRGVRA
AN0576.1  	QAKGISDKEIARRSPPNLKDYEAWHVEGDRPEGMLS----RFGTGTVEPPSGSPSTGTSS
          	.:.. :... :  .    :.*.**.*: *: ..  .       *.      .... *. :

MG06100.1 	MVVGS---SSASVEVDNGGGGGGRDIRYAYILTAGSDKKLRFWDLSRIENSTVFSGIGPE
NCU06626.1	LVFGWGITDAATVEEQPPPPLQERDVRYAFMVTGGSDKKLRFWDLTHVSQSRIYSGLQLG
FG05306.1 	MVVGT-----ATVD-------DSRDVRHAFIVTGGSDKRLRFWDLSRMESSFIYSGLTPD
AN0576.1  	GTGIN----TFIGGFDCPEDGRDNSTRVGFIISGGCDRKIRFWDLHRPDQSCIISGLDPI
          	 .       :     .       .. * .::::.*.*:::***** : ..* : **:   

MG06100.1 	EP-----RPSFTASHPTTALTLNTERLPRQSVG----AAGDDGKGKG-------------
NCU06626.1	EA-----APSYTASHPTPSLILNTERLHGKSSGDQASGSGVTGTGRGSGVAQGVNGKSSP
FG05306.1 	QA-----KPTYTTSHPTTALALNMERFPRQAPTAPNAGSGSKAKSAS-------------
AN0576.1  	SDGTVTGSPRYEVSSPTQSLTFAIEHLPNPGAN----AGGAKGSGKRSG-----------
          	. .: :. * : .* ** :* :  *::   .      ..*  ...  :.           

MG06100.1 	----------RAKTARSTVVSIQQQQLLRSHLDAVLDVAVIESPYTMTVSVDRMGVVFVF
NCU06626.1	SSNSARNAQQKMRPSRTKIISQEHFRLLRSHLDSVLDVALLESPYPMTVSCDRGGMVLII
FG05306.1 	-----------ARPPRSTVISLQQQQLLQSHMDSILDVALLEFPYTMSVSVDRMGVMFVF
AN0576.1  	----------GGRLPRSTVISLQQQQLLKSHLDFIQDIAVLRVPYGMIISVDRAGMVYVF
          	            : .*:.::* :: :**:**:* : *:*::. ** * :* ** *:: ::

MG06100.1 	Q
NCU06626.1	R
FG05306.1 	Q
AN0576.1  	Q
          	:
```
